# The Role of Social Support and Socioeconomic Factors in the Prediction of Depression among First-Year Undergraduate Students

**DOI:** 10.1155/2023/3993304

**Published:** 2023-08-05

**Authors:** Razieh Sadeghi, Maedeh Ghandehari Alavijeh, Hadi Raeisi Shahraki

**Affiliations:** ^1^Student Research Committee, Shahrekord University of Medical Sciences, Shahrekord, Iran; ^2^Department of Epidemiology and Biostatistics, Faculty of Health, Shahrekord University of Medical Sciences, Shahrekord, Iran; ^3^Modeling in Health Research Center, Shahrekord University of Medical Sciences, Shahrekord, Iran

## Abstract

**Aim:**

This study was devoted to determining the role of social support and socioeconomic factors in predicting students' depression.

**Methods:**

In this cross-sectional study, all first-year undergraduate students in the Shahrekord University of Medical Sciences, Iran, during the 2019-2020 academic year were included via the census method. Data collection tools include a researcher-made checklist about demographic and socioeconomic status, a standard questionnaire of perceived social support, and Beck's depression questionnaire. Smoothly clipped absolute deviation (SCAD) linear regression was used to model the role of social support and socioeconomic factors in predicting depression.

**Results:**

Out of the 220 first-year undergraduate students, 174 (79.1%) were female, and 176 (80.0%) were single. The mean ± SD of depression score among the first-year undergraduate students was 10.56 ± 5.19, and the mean ± SD of social support score was 48.86 ± 5.46. The mean score of depression was significantly higher in female students than in males (11.09 versus 8.59, *P* = 0.001) but was not statistically significant in different categories of age (*P* = 0.70), marital status (*P* = 0.37), ethnicity (*P* = 0.10), parents' education, and the other demographic variables. Pearson's correlation showed an inverse and significant correlation between depression and social support (*R* = −0.20, *P* = 0.003). The mean score of depression was at the highest level for students of public health and environmental health majors and was the lowest for students of laboratory sciences, which was statistically significant (*P* < 0.001). After adjusting the other variables, SCAD regression showed that social support plays a key role in depression prediction, and increasing social support leads to a decrease in depression score.

**Conclusion:**

Considering the existence of an inverse and significant correlation between depression and social support, any intervention to promote social support for first-year undergraduate students may decrease depression.

## 1. Introduction

Undergraduate students are known as one of the most vulnerable groups, and associated factors with their mental health should be considered. Entering the university environment leads to significant challenges for undergraduate students and increases the odds of depression [1, 2]. Depression is one of the most common mental illnesses with the most common symptoms of feelings of guilt, anorexia, decreased energy and interest, difficulty concentrating, thoughts of death, and suicide. Depression affects both women and men and is not limited to a specific place, time, or country [3–5]. Depression and anxiety disorders decrease the level of mental health of students [6]. There are various factors involved in depression, the most important of which are social, cultural, and psychological factors [7, 8]. The 2019 coronavirus disease (COVID-19) has resulted in adverse consequences such as a global health crisis. This disease is one of the factors that has had consequences on the mental health of students and has caused an increase in anxiety and depression in them [9]. In addition, today, one of the most important factors that cause health problems such as depression, anxiety, psychiatric disorders, and low self-esteem, especially among students, is inappropriate and excessive use of the Internet. Therefore, it is necessary to design and implement appropriate educational programs to promote healthy behavior in students [10].

Social support is one of the other factors that affect students' mental health. A reasonably comprehensive definition of social support found in many studies is the feeling that others love and value the person and are willing to help and emotional support [11]. Most researchers in the field of stress management pay attention to the social support factor as one of the factors that can prevent the occurrence of stress-related diseases. People who receive better social support have lived more coherent, and they experience less stress and depression throughout their lives [12]. According to previous studies, there is an inverse association between the amount of social and family support received by students and their level of depression by increasing students' self-esteem [13]. Moreover, the socioeconomic level of students and their parents may be associated with the mental health of undergraduate students.

Since the proportion of depression is high in students studying in the first or second year of study and performed studies that have addressed this issue are rare, this study was devoted to determining the role of social support and socioeconomic factors in predicting students' depression.

## 2. Materials and Methods

In this cross-sectional study, all first-year undergraduate students in the Shahrekord University of Medical Sciences (SKUMS) in the 2019-2020 academic year were included via the census method, and incomplete questionnaires were excluded. By visiting the place of holding classes, participants were informed about the purpose of the study, and questionnaires were distributed among those who were willing to participate. Data collection tools include a researcher-made checklist about demographic and socioeconomic status, a standard questionnaire of perceived social support, and Beck's depression questionnaire.

The perceived social support questionnaire was developed by Zimet et al. to assess social support using 12 questions from three essential sources of family, friends, and others. The total score of the questionnaire ranged from 12 to 60, and higher scores indicate a higher level of perceived social support. Also, the reliability of the questionnaire by Cronbach's alpha method was mentioned as 0.83 [14].

Beck's depression questionnaire included 21 questions, which Beck and colleagues first developed in 1961. Each of the 21 questions dealt with one of the symptoms of depression, and the total score ranged from 0 to 62. The validity of the Persian version of Beck's depression questionnaire has been approved in the previous studies. The reliability of the questionnaire has also been reported by Cronbach's alpha 0.93 [15, 16].

Descriptive findings were presented in number (%) and mean ± SD for qualitative and quantitative variables, respectively. Moreover, bivariate analysis was performed using Pearson's correlation, ANOVA, and independent *T*-test in SPSS 21 software. Smoothly clipped absolute deviation (SCAD) linear regression was used to model the role of social support and socioeconomic factors in predicting depression among undergraduate students. Compared to traditional variable selection techniques, SCAD performs simultaneous estimation and variable selection and has numerous advantages [17, 18]. The SCAD regression was performed in *ncvreg* package in R 3.5.3 software. To achieve a reliable finding, the 5-fold cross-validation was performed 1001 times and the median of the obtained tuning parameter was reported as the optimum tuning parameter. Moreover, to estimate the standard errors, 1000 bootstrap samples were generated and the standard deviations of coefficients were calculated based on bootstrap samples.

This study was approved by the Ethics Committee of SKUMS (ethics code: IR.SKUMS.REC.1398.152). All subjects voluntarily participated and were assured that all information would remain confidential.

## 3. Results

Out of the 220 first-year undergraduate students, 174 (79.1%) were female, and 176 (80.0%) were single. In terms of ethnicity, most of the participants (47.7%) were Fars and in the age range of 20-21 years (57.3%). Although 136 (61.8%) students had a private laptop, just 41 (18.6) had a private car. The mean ± SD of depression score among the first-year undergraduate students was 10.56 ± 5.19, and the mean ± SD of social support score was 48.86 ± 5.46. The mean score of depression was significantly higher in female students than in males (11.09 versus 8.59, *P* = 0.001), but depression scores were not statistically significant in different categories of age (*P* = 0.70), marital status (*P* = 0.37), ethnicity (*P* = 0.10), parents' education, and the other demographic variables (Table 1).

**Figure 1:**
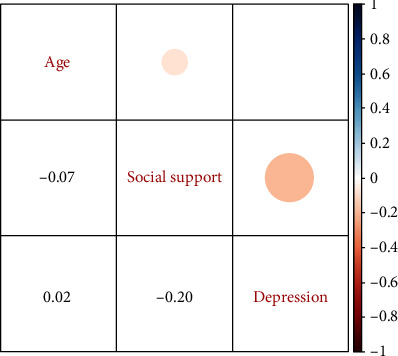
The Pearson correlation between age, social support, and depression.

Pearson's correlation showed an inverse and significant correlation between depression and social support as an increase in social support was associated with a decrease in depression score (*R* = −0.20, *P* = 0.003, Figure 1). The mean score of depression was at the highest level for students of public health and environmental health majors and was at the lowest level for students of laboratory sciences, which was statistically significant (*P* < 0.001). On the other hand, students in laboratory sciences and midwifery majors had the highest and the lowest scores in social support, respectively (Table 2).

SCAD regression showed that even after age, father and mother education, and significant adjustment, social support plays a key role in depression prediction, and increasing social support leads to a decrease in depression score (Table 3).

## 4. Discussion

Our study showed a significant difference between the level of depression in male and female students so that the rate of depression in female students was higher than in male students. This finding is in line with the study of Hamaideh and Maroufi study and colleagues [19, 20]. Similarly, in the study of Taziki et al. and the survey of Karami, female students were significantly more depressed than male students which may be due to the genetic and hormonal differences between girls and boys [21, 22].

Inconsistent with the findings of our study, Lenzo et al. showed a significant and negative correlation between social support and depression [23, 24]. To justify this correlation, it can be said that social support is one of the most vital forces for coping successfully and efficiently with difficult situations and stressful situations and makes it easier for people to endure problems and maintain peace [23, 25]. Suarez and Ramirez believe that social support enhances the quality of life, improving the economic situation maintaining social cohesion, bonding with society, and dealing with loneliness [26]. According to Maleki et al. findings, social support on behalf of friends is directly related to depressive symptoms in students, and social support on behalf of a family is indirectly associated with depressive symptoms in students. Social support gives students a sense of worth and self-esteem and reduces depression [27, 28].

The results of our study showed that the level of social support and depression was significantly different among students of different majors. Depression scores of laboratory science students were much lower than the rest, and environmental and public health students were much higher than other students. This finding is entirely inconsistent with the results of Hadavi and Rostami [29] and Saki and Kaikhavani [30]. According to Hadavi and Rostami, the highest frequency of depression was in laboratory science students (44%), and the lowest frequency of depression was in midwifery students (24%). This difference may be due to the selection of all the students regardless of degree and semester [29]. Also, the study of Saki showed that the rate of depression in medical, laboratory science, anesthesiology, and nursing students was higher than in other fields, which can be due to expectations beyond the existing facts and worries about the future career [30].

The frequency of mental disorder symptoms among the students has been investigated in the study of Taziki et al. They showed that symptoms of depression and mental disorders were significantly higher in single people than in married people [22]. Our study showed that the score of depression in married students was lower than in single people, but no significant difference was observed. Moreover, according to research by the US Department of Public Health, married people are significantly better off in terms of mental health than single people, and a lower percentage of them suffer from physical and mental illness [31]. According to the findings of Danesh, the symptoms of anxiety, depression, and social dysfunction in married students are less than in single students [32]. Gove and Hughes also reported that the strength of marital relationships contributes significantly to the mental health of married people compared to single people [33].

Our study showed that the depression scores of students whose fathers were illiterate were lower than others, but no significant difference was observed. This finding is not consistent with the study of Jahangasht et al. and Molabaghery et al., which showed that there is a significant inverse relationship between adolescent depression and parents' education; that is, the lower the parents' education, the greater the symptoms of adolescent depression. Accordingly, parents with higher levels of education have a greater understanding of their children's educational status and potentially related problems [34, 35]. Hashemi et al. believed that parents' education had no statistically significant relationship with the rate of depression [36]. On the other hand, Salajegheh et al. found that a high level of education of the mother improves the ability to understand mental states and interaction with children and reduces the risk of depression and mental disorders in children [37].

According to our findings, the depression scores of students who had a personal car or laptop were lower than the others, but no significant difference was observed. Also, the depression score of students with personal income was lower, but no significant difference was observed. This finding is consistent with the study of Rezapour et al., which showed that students' economic status and income could not predict mental health [38]. Another finding in this study was that depression scores were lower in students with higher household incomes, but no significant difference was observed. This finding is in line with the results of some studies [34, 39]. In this regard, Shamsuddin et al. found that stress and depression were higher in students with low- or high-income families than in those with middle-income families [40]. Also, Rezapour et al. have reported a significant difference between families in terms of different levels of economy and income; in this way, people from low-income families are more depressed than people from high-income families. Unfavorable economic conditions affect people's moods and create stress in them, and in such circumstances, a person feels depressed. In general, depression is more common in weak socioeconomic classes [38].

From the academic purposes point of view, our study showed that the level of depression is strongly correlated with the level of perceived social support by undergraduate students. Due to the fact that mean score of depression was significantly higher in female students, it is critical to improve the level of perceived social support especially in female students. In the present study, the association between four important psychological variables and some socioeconomic variables was investigated. Our research population was limited to the eight majors in SKUMS, and we did not consider undergraduate students in the other majors. Also, the existence of uncontrolled variables such as lifestyle and educational condition of students up to the entrance exam may confound its generalizability.

## 5. Conclusion

Considering the existence of an inverse and significant correlation between depression and social support, any intervention to promote the social support of first-year undergraduate students may decrease depression.

## Figures and Tables

**Table 1:** Demographic characteristics of the first-year undergraduate students.

Variable	Subgroup	Number (%)	Mean ± SD of depression	*P* value
Gender	Female	174 (79.1)	11.09 ± 5.34	0.001
	Male	46 (20.9)	8.59 ± 4.03	

Age	18-19	94 (42.7)	10.40 ± 4.46	0.70
	20-21	126 (57.3)	10.68 ± 5.69	

Marital status	Single	176 (80.0)	10.72 ± 5.22	0.37
	Married	44 (20.0)	9.93 ± 5.05	

Ethnicity	Fars	105 (47.7)	9.79 ± 5.00	0.10
	Lor	87 (39.5)	11.21 ± 5.24	
	Other	28 (12.7)	11.46 ± 5.49	

Current residency	Dormitory	176 (80.0)	10.47 ± 4.86	0.60
	With family	44 (20.0)	10.93 ± 6.38	

Father education	Illiterate	35 (15.9)	8.80 ± 4.82	0.09
	< dip	104 (47.3)	10.90 ± 5.12	
	> dip	81 (36.8)	10.89 ± 5.33	

Mother education	Illiterate	26 (11.8)	10.27 ± 5.19	0.71
	< dip	116 (52.7)	10.84 ± 5.20	
	> dip	78 (35.5)	10.26 ± 5.21	

Having private laptop	No	84 (38.2)	10.77 ± 4.95	0.34
	Yes	136 (61.8)	10.43 ± 5.34	

Having private car	No	179 (81.4)	10.89 ± 5.15	0.06
	Yes	41 (18.6)	9.15 ± 5.16	

Having income	No	140 (63.6)	10.76 ± 4.91	0.47
	Yes	80 (36.4)	10.23 ± 5.66	

Family income	<$200	21 (9.6)	11.81 ± 5.96	0.48
	$200-$250	115 (52.3)	10.55 ± 4.99	
	>$250	84 (38.2)	10.27 ± 5.27	

**Table 2:** Comparison of depression and social support among different majors.

Major	Mean ± SD of depression	*P* value	Mean ± SD of social support	*P* value
Nursing (*n* = 50)	8.44 ± 4.28	<0.001	48.74 ± 5.82	<0.001
Midwifery (*n* = 24)	12.00 ± 5.78		46.50 ± 5.11	
Surgical technology (*n* = 26)	8.92 ± 3.50		48.46 ± 5.20	
Anesthesia (*n* = 23)	11.48 ± 1.86		45.91 ± 4.80	
Radiologic technology (*n* = 19)	9.74 ± 3.43		47.16 ± 6.05	
Laboratory sciences (*n* = 24)	4.92 ± 1.84		54.63 ± 2.14	
Environmental health (*n* = 27)	14.70 ± 4.84		49.67 ± 4.57	
Public health (*n* = 27)	15.48 ± 5.15		49.33 ± 4.60	

**Table 3:** Summary of SCAD linear regression model.

Variable	Subgroup	Coefficient	SE
Age	18-19	Baseline	—
	20-21	0.20	0.56

Father education	Illiterate	Baseline	—
	< dip	1.70	1.28
	> dip	2.13	1.62

Mother education	Illiterate	Baseline	—
	< dip	0.00	0.77
	> dip	-0.37	1.18

Major	Laboratory sciences	Baseline	—
	Nursing	3.12	1.35
	Midwifery	6.64	1.85
	Surgical technology	3.35	1.59
	Anesthesia	6.03	1.59
	Radiologic technology	4.31	1.68
	Environmental health	9.47	1.44
	Public health	10.03	1.44

Social support		-0.05	0.08

Dependent variable is depression score.

## Data Availability

The data used to support the findings of this study are available from the corresponding author upon request.
